# Nijmegen Breakage Syndrome Detected by Newborn Screening for T Cell Receptor Excision Circles (TRECs)

**DOI:** 10.1007/s10875-015-0136-6

**Published:** 2015-02-13

**Authors:** Jay P. Patel, Jennifer M. Puck, Rajgopal Srinivasan, Christina Brown, Uma Sunderam, Kunal Kundu, Steven E. Brenner, Richard A. Gatti, Joseph A. Church

**Affiliations:** 1 Division of General Pediatrics, Children’s Hospital of Los Angeles, Los Angeles, CA USA; 2 Department of Pediatrics and Institute for Human Genetics, University of California, San Francisco and UCSF Benioff Children’s Hospital, San Francisco, CA USA; 3 Tata Consultancy Services Ltd., Hyderabad, Telengana India; 4 Departments of Human Genetics and Pathology & Laboratory Medicine, David Geffen School of Medicine, University of California, Los Angeles, CA USA; 5 Department of Plant and Microbial Biology, University of California, Berkeley, CA USA; 6 Division of Clinical Immunology and Allergy, Children’s Hospital of Los Angeles, Keck School of Medicine, University of Southern California, Los Angeles, CA USA

**Keywords:** Nijmegen breakage syndrome, TREC, SCID, exome sequencing, nibrin, T lymphopenia

## Abstract

**Purpose:**

Severe combined immunodeficiency (SCID) encompasses a group of disorders characterized by reduced or absent T-cell number and function and identified by newborn screening utilizing T-cell receptor excision circles (TRECs). This screening has also identified infants with T lymphopenia who lack mutations in typical SCID genes. We report an infant with low TRECs and non-SCID T lymphopenia, who proved upon whole exome sequencing to have Nijmegen breakage syndrome (NBS).

**Methods:**

Exome sequencing of DNA from the infant and his parents was performed. Genomic analysis revealed deleterious variants in the *NBN* gene. Confirmatory testing included Sanger sequencing and immunoblotting and radiosensitivity testing of patient lymphocytes.

**Results:**

Two novel nonsense mutations in *NBN* were identified in genomic DNA from the family. Immunoblotting showed absence of nibrin protein. A colony survival assay demonstrated radiosensitivity comparable to patients with ataxia telangiectasia.

**Conclusions:**

Although TREC screening was developed to identify newborns with SCID, it has also identified T lymphopenic disorders that may not otherwise be diagnosed until later in life. Timely identification of an infant with T lymphopenia allowed for prompt pursuit of underlying etiology, making possible a diagnosis of NBS, genetic counseling, and early intervention to minimize complications.

## Introduction

Severe combined immunodeficiency (SCID) refers to a group of life-threatening fatal, congenital disorders characterized by absent or reduced T-cell number and reduced or non-functional B-cells. An early diagnosis of SCID allows for avoidance of exposure to infections, live virus vaccines and non-irradiated blood products, and also makes possible early hematopoietic stem cell transplantation (HSCT) or other immune system restoring treatments, adenosine deaminase enzyme replacement, or gene therapy [1].

Since beginning in 2008 in Wisconsin, newborn screening for SCID has been implemented in 23 states in the U.S., as well as in the Navajo Nation [2]. The assay employs PCR quantification of circular DNA byproducts of T-cell receptor gene rearrangement, T-cell receptor excision circles (TRECs). Although TRECs have no identifiable function, their presence serves as a marker for the maturation of T-cells [3]. Insufficiency of TRECs characterizes SCID and other T-lymphopenic disorders including complete DiGeorge Syndrome [4] and some cases of ataxia telangiectasia (AT) [5].

Like AT, Nijmegen breakage syndrome (NBS) [6–9] (formerly known as an AT Variant 1) is a disorder of DNA repair associated with chromosomal instability and immune compromise that may be identified through TREC screening. Herein, we describe a baby with a complex perinatal history and low TRECs near birth. Flow cytometry confirmed modest T-lymphopenia. Whole exome sequencing identified heterozygous mutations in *NBN*, the gene that encodes nibrin, a component of a molecular complex involved in the early recognition and subsequent repair of DNA damage [10]. Nibrin was absent from nuclear lysates on immunoblots, and the patient’s cells were hypersensitive to ionizing radiation by colony survival assay [11, 12].

## Case Report

A 41-week gestational age male (NBS20LA), weighing 2860 grams, was born via normal vaginal delivery to a 23-year-old, gravida 1, paragravida 0 mother following prenatal identification of increased cerebrospinal fluid on ultrasound. Postnatally, the infant was hospitalized for 1 week for meconium aspiration. By 4 weeks of age progressive hydrocephalus required a ventriculo-peritoneal shunt. At 17 weeks, he was hospitalized for a presumed urinary tract infection; renal ultrasound showed left hydronephrosis. During the first year of life he also exhibited developmental delay and failure to thrive with weight and length for age persistently below the 3rd percentiles and head circumference at 8 months of age below the 2nd percentile.

At 3 weeks of age, SCID newborn screening results were interpreted as “incomplete”: TREC, 13 copies/mcL (normal >25) and actin control, 9,510 copies/mcL (normal >10,000). Repeat screening was “positive”: TREC, 12 copies/mcL and actin, 19,800 copies/mcL. Subsequent flow cytometry at 6 weeks of life demonstrated persistent T-lymphopenia and elevated NK cell percentages and numbers (Table 1).

**Table 1:** Laboratory values of patient NBS20LA from 1.5 months to 1 year of age

Patient Age	1.5 month	3 months	5.5 months	10 months	Reference Ranges		
					Birth to 2 months	2 months to 4 months	4 months to 1 year
Total WBC cells/uL	6800	5120	5500	7880	5000–17,000	5000–17,000	5000–17,000
%Neutrophils	47	44	58	56	25–45	25–45	25–45
%Lymphocytes	33	27	23	22	46–66	46–66	46–66
%Monocytes	8	19	12	14	2–8 %	2–8 %	2–8 %
%Eosinophils	12	10	7	8	0–3	0–3	0–3
CD 3+ T cells/mL (%)	924 (42)	259 (24)	289 (18)	381 (22)	1375–7129 (69–93)	2533–6778 (55–79)	2889–7995 (59–80)
CD 4+ T cells/mL (%)	660 (30)	65 (5)	224 (15.1)	320 (19)	1169–5623 (48–75)	1392–5210 (35–62)	1786–5141 (38–61)
CD 8+ T cells/mL (%)	242 (11)	99 (8)	63 (4)	72 (4)	267–1860 (13–33)	652–2449 (14–30)	946–2791 (14–34)
CD19+ B-cells/mL (%)	88 (4)	26 (2)	72 (5)	174 (10)	104–1448 (4–26)	745–3499 (14–39)	858–3774 (16–36)
CD3-CD16/56+ NK cells /mm3 (%)	638 (29)	680 (66)	1123 (73)	1169 (68)	60–434 (2–14)	194–994 (3–14)	182–1581 (3–13)
IgG (mg/dL) [Reference Range]		220 [198–577]	202 [165–781]	316 [282–1026]			
IgM (mg/dL) [Reference Range]		24 [16–100]	34 [31–103]	70 [39–142]			
IgA (mg/dL) [Reference Range]		<7 [1–52]	7 [8–83]	16 [16–83]			
Ab Responses:							
Tetanus (IU/mL) (RR: 0.1–7)			2.19	2.71			
H. influenzae b (mcg/mL) (RR: 1.0–10.0)		0.15	1.16				
LPA responses:							
Phytohemaglutinin (SI) [1:125]		173	44	42			
Pokeweed mitogen (SI)		23	75	23			
Tetanus antigen (SI)			2	1			
Candida antigen (SI)			4	6			

Targeted sequencing of SCID associated genes *ADA*, *AK2*, *CD3D*, *CD3zeta*, *DCLRE1C*, *ILRG*, *IL7R*, *JAK3*, *LIG4*, *NHEJ1*, *PNP*, *PTPRC*, *RAC2*, *RAG1*, *RAG2*, *RMRP*, and *ZAP70* (GeneDx, Gaithersburg, MD) and enzyme assays for adenosine deaminase and nucleoside phosphorylase revealed no abnormalities.

## Methods

### Subjects and Samples

Informed consent was obtained for research, including cellular immune studies and whole exome sequencing (WES), for the infant and both parents under approved protocols at Children’s Hospital Los Angeles (CHLA) and the University of California San Francisco (UCSF). Genomic DNA from EDTA-anticoagulated whole blood was prepared using a Gentra Puregene Blood kit (Qiagen USA: Germantown, MD).

### Exome Sequencing and Analysis

WES was performed as previously described [5]. Briefly, libraries prepared by ligating TruSeq adaptors (Illumina: San Diego, CA) to genomic DNA fragments of 200–300 bp were enriched with 10 cycles of PCR, pooled and submitted to exon capture using a Roche Nimblegen version 3.0 capture array. After 10 additional amplification cycles, 100 bp paired end sequence reads were generated (HiSeq2000, llumina), yielding 3.8 % duplicates and a mean of >100 reads covering the targeted regions, with >95 % of target regions having ≥10 reads.

Reads were aligned against GRCh37 (Aug 2009 release) using BWA (v0.6.2) [13]. The results were converted to BAM, sorted by coordinate, indexed, and marked for PCR duplicate reads using the Picard toolkit (v 1.81) (http://picard.sourceforge.net). Local realignment was performed around known indel locations, and base quality scores were re-calibrated using GATK (v 2.6.5) [14, 15].

Variants were called using GATK UnifiedGenotyper and freebayes (vesion 0.9.10) [16]. GATK variant quality scores were re-calibrated by VQSR (Variant Quality Score Recalibration) using the trio of exomes in this report, as well as 65 others sequenced at our site. HapMap v3.3 [17], 1000 genomes high confidence SNPs (phase1 v3 2010-11-23) and Omni chip array sets were used as training data, and to provided truth sites for SNPs, while the Mills dataset [18] from the 1000 genomes was used for indels, using a truth sensitivity cutoff of 99 %. Variant annotation (including region, effect, allele frequency, disease phenotype annotation, and conservation) was performed using our custom tool Varant (http://compbio.berkeley.edu/proj/varant/).

Particular attention was given to high-confidence, rare, likely-damaging, protein-altering variants in genes associated with primary cellular immunodeficiency [19] and to those conforming to models of autosomal or X-linked recessive inheritance in the family, or *de novo* mutations that may be dominant. Candidate variants in *NBN* were given priority and confirmed by Sanger sequencing.

### Immunoblotting

Peripheral blood lymphoblastoid cell lines (LCL) from the patient NBS20LA and controls were made with Epstein-Barr virus as described [11], and 50 μg of nuclear protein lysate from each was electrophoresed on a 6 % SDS-polyacrylamide gel (PAGE), blotted onto PVDF membrane (BioRad, Hercules, CA) and incubated with antibodies to nibrin (Novus, NB100-143 at 1:5000, Littleton, CO) overnight at 4 °C. The immunoblots were subsequently incubated with an HRP-conjugated anti-rabbit secondary antibody at room temperature for 40 min for detection by enhanced chemiluminescence (ECL) (Amersham Pharmacia, Piscataway, NJ). Under the conditions of the assay, as little as 1 μg of nibrin protein could be detected. p84 was used as a loading control (Genetex GTX70220 at 1:3000). On the same blot, MRE11 and Rad50 were also measured (Novus 100–142 at 1:15,000 and Novus 100–154 at 1:500, respectively).

Peripheral blood lymphocytes from the patient NBS20LA and controls were immortalized with Epstein-Barr virus as previously described [11], and 50 μg of nuclear lysate protein from the each lymphoblastoid cell line (LCL) was electrophoresed on a 6 % SDS-polyacrylamide gel (PAGE), blotted onto PVDF membrane (BioRad, Hercules, CA) and incubated with antibodies to nibrin and DNA repair proteins MRE11, RAD50 and ATM (Novus, Littleton, CO) overnight at 4 °C. The immunoblots were subsequently incubated with an HRP-conjugated anti-rabbit secondary antibody at room temperature for 40 min for detection by enhanced chemiluminescence (ECL) (Amersham Pharmacia, Piscataway, NJ). Under the conditions of the assay, as little as 1 μg of nibrin protein can be detected.

### Colony Survival Assay

LCLs from wild type (healthy controls), individuals with AT (radiosensitive controls), and NBS20LA were maintained in RMPI 1640 medium with 10 % fetal bovine serum and 1 % penicillin-streptomycin-L-glutamine [12]. After plating into paired 96-well trays, one of each pair was irradiated with 1 Gray. After incubation at 37 °C for 2 weeks, the trays were stained with 0.1 % 3-(4,5-dimethylthiazol-2-yl)-2,5-diphenyltetrazolium bromide. Individual wells with at least one colony composed of >32 cells (i.e., >5 divisions) were scored as positive. The number of positive wells in irradiated- versus non-irradiated plates was compared to determine the survival fraction. Results were compared to measurements performed by Sun et al. using 104 AT patients and 29 healthy controls [12].

## Results

WES analysis by our Varant tool identified two nonsense mutations in *NBN* on chromosome 8q21.3 in the patient, suggesting a diagnosis of NBS. The patient and his father shared a thymine to guanine transversion cDNA 842 T > G (NM_002485.4(NBN):c.842 T > G) in exon 6, changing leucine 281 to a stop codon (protein L281X) (ClinVar Provisional Accession: SCV000196640), while the infant and mother shared a cytosine to thymine transition cDNA 1030C > T (NM_002485.4(NBN):c.1030C > T) in exon 9, changing glutamine 344 to a stop codon (Q344X) (ClinVar Provisional Accession: SCV000196641) [20]. A rare X-linked *CD40LG* gene variant noted in our patient and mother NM_000074 (CD40LG):c.542G > C (chrX: 135741330, G > C; R181P in CD40LG) was predicted damaging by PolyPhen-2 software [21], but was inconsistent with the patient’s phenotoype. No other high quality DNA variants in the targeted exome regions were as consistent with the phenotype. Neither variant had been reported in other patients with NBS nor in the 1000 genomes [22], ESPDB [23] or dbSNP (v137) [24] databases. The patient’s paternally inherited allele carried L281X, while his maternally inherited allele carried Q344X. Confirmatory Sanger sequencing was performed by the CLIA-approved Molecular Genetics Laboratory at the University of California Los Angeles School of Medicine.

**Fig. 1:**
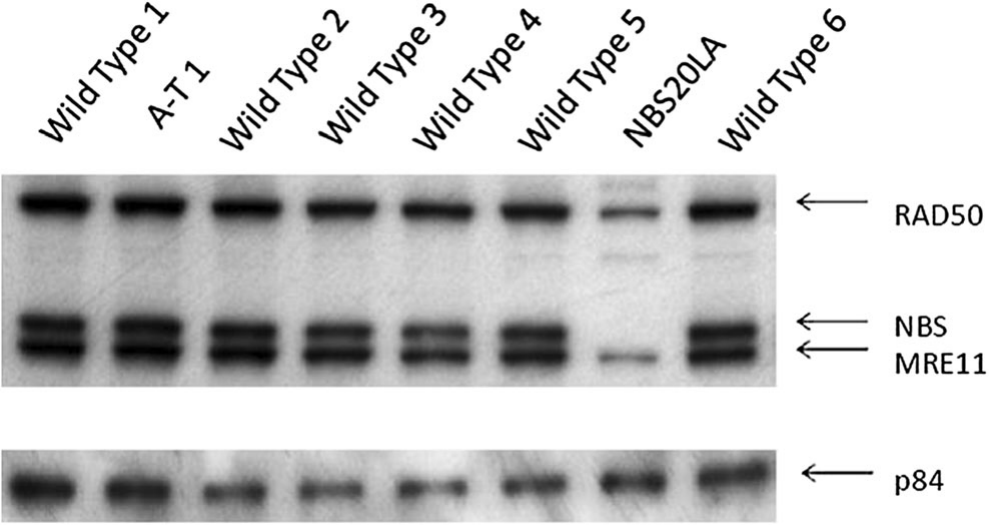
Immunoblot allows comparison of nibrin (NBS), MRE11 and Rad50 protein levels in an NBS cell line (NBS20LA) to wild type and AT cells. Note absence of nibrin in NBS LCL. Protein loading control was p84

**Fig. 2:**
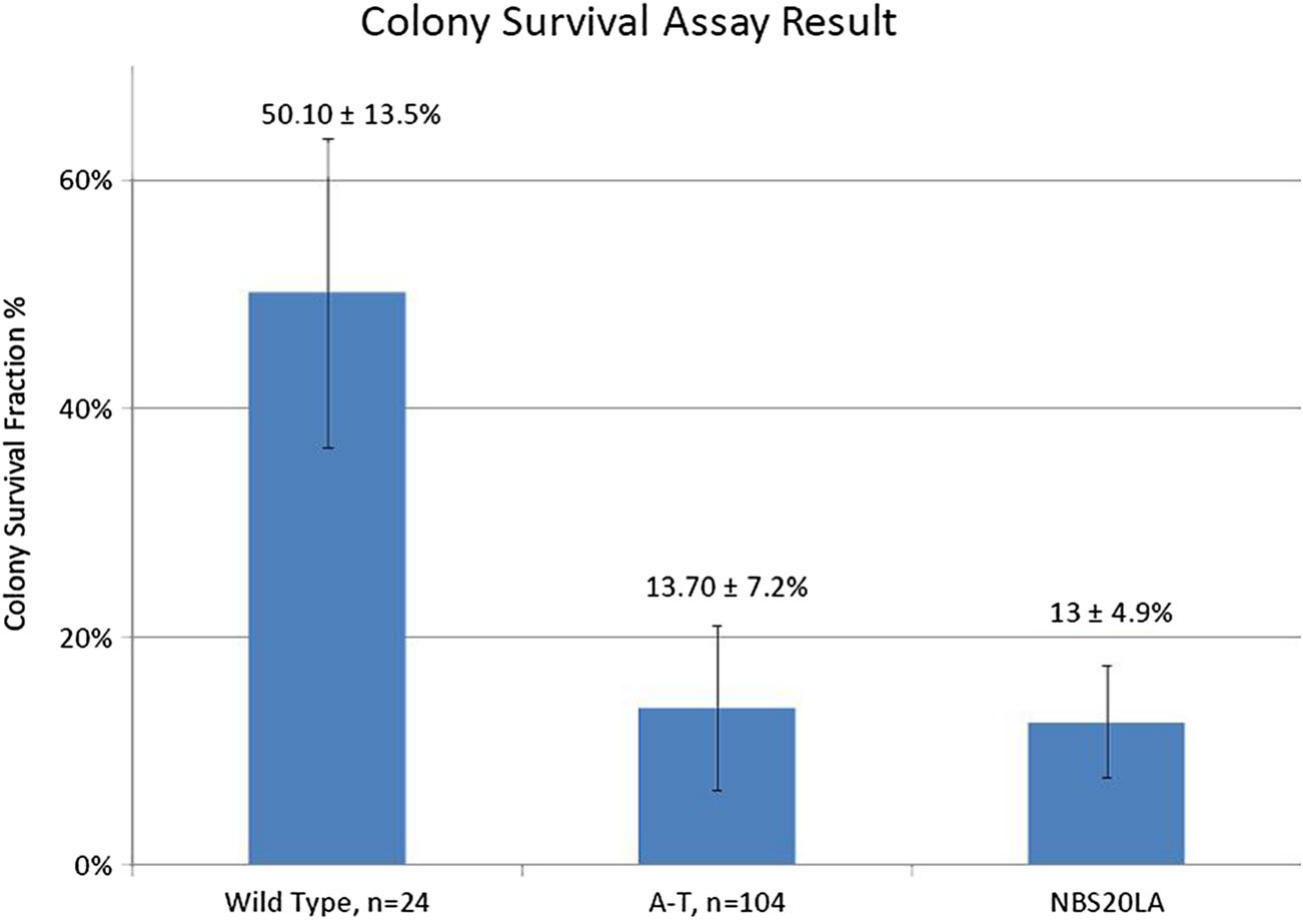
Colony Survival Assay for AT and NBS B lymphoblastoid cell lines showing that both the AT control cells and those of patient NBS20LA are in the radiosensitive range, as defined by Sun et al. 2002 [12]

An immunoblot showed no detectable nibrin protein in NBS20LA (Fig. 1, penultimate lane). Interestingly, MRE11 and Rad50 protein levels were also reduced; this reflects the instability of the MRE11-Rad50-nibrin (MRN) complex whenever nibrin is absent. Lane 2 contains LCL protein from an AT patient whose cell lines were radiosensitive in Fig. 2. ATM protein was absent in the ATM LCL, but was present in NBS20LA (not shown). All other LCLs in Fig. 1 were from healthy individuals and showed normal amounts of MRN-complex proteins and the loading control, p84. These findings provided functional confirmation that the patient’s *NBN* variants were both null mutations that abrogated protein expression, and that his cells scored as radiosensitive.

## Discussion

Although the primary goal of newborn screening with TRECs has been the pre-symptomatic diagnosis of SCID, thereby allowing early intervention, this screening has also identified individuals with non-SCID T-lymphopenia. In California, the first 2 years of newborn screening for SCID identified 50 infants with T-lymphopenia, defined as <1500 T cells/mcL: 11 had typical SCID, five had idiopathic T-lymphopenia or variant SCID (defined as significant T cell defects without mutation in known SCID genes), eight had partial and one had complete DiGeorge syndrome, four had trisomy 21, and three had AT [5]. Twenty-three patients had preterm birth or other congenital anomalies [4].

Recently, exome sequencing was utilized to identify AT as the cause of T-lymphopenia in two patients with positive SCID screens [5]. In this same study retrospective analysis of older AT patients suggested that approximately half of patients with AT will have a positive newborn SCID screen. NBS is a distinct DNA breakage/chromosomal instability disorder caused by defects in nibrin, part of the same DNA repair pathway as AT mutated protein. The immune defects in AT and NBS are similar and include impaired VDJ recombination, which affects T-cell receptor and immunoglobulin rearrangement. T-lymphopenia and decreased TRECs as seen in patients with AT would thus also be expected in patients with NBS.

In our patient, the TREC newborn screening for SCID was positive, but an extensive search for SCID gene mutations was unrevealing. In contrast, exome sequencing revealed novel, compound heterozygous *NBN* mutations that were associated with absence of nibrin and radiosensitivity. A R181P missense variant in *CD40LG*, the gene associated with X-linked hyper-IgM syndrome, was also predicted to be damaging but was inconsistent with the patient’s clinical and laboratory data including absent nibrin and a total serum IgG concentration in the normal range and positive IgG antibody responses to tetanus and *H. influenzae b*. Although it is not impossible for a patient to have two primary immune disorders, *NBN* mutations fully explain the low T cells, absent nibrin and cellular radiosensitivity that establish the diagnosis of NBS in this patient, while the features of CD40L deficient hyper-IgM syndrome are absent.

The clinical phenotype of NBS is variable. Most patients are full term [7], but may have microcephaly at birth with subsequent growth failure. Most patients meet early developmental milestones but have varying degrees of intellectual disability later in life [7, 25–27]. Perinatal hydrocephalus as seen in our patient has been reported in two children with NBS [8]. Hydronephrosis, renal anomalies such as horseshoe or dysplastic kidneys, skeletal anomalies such as clinodactyly and partial syndactyly, café au lait spots and vitiligo have all been seen, but are not constant features and are also found in other conditions [8, 28]. It is therefore difficult to identify NBS based on early clinical signs. Lymphoid malignancy affects up to 40 % of patients with NBS by their 21st year [28]. Onset of malignancy can vary in these patients depending on the mutation inherited and degree of nibrin dysfunction [28].

Most patients with NBS show some degree of immunodeficiency. In a review of 57 patients from the Polish Nijmegen Breakage Syndrome Registry, 19 % had normal total serum immunoglobulins, while 25 % had severe hypogammaglobulinemia [29]. At least one IgG subclass was deficient in all patients: 89 % IgG4, 73 % IgG2 and 54 % IgG1. Similarly, our patient had variably low total serum immunoglobulins that trended upward with age (Table 1), but IgG3 and IgG4 remained low (data not shown). Specific antibody response after two *H. influenza* type b immunizations was negative at 6 months, but positive at 10 months following an additional immunization. Tetanus toxoid titers were positive by 6 months. Our patient’s absolute CD19+ B cells were reduced during the first year of life, as seen in 72 % of Polish registry patients [29]. In the Polish cohort, 84 % of patients received immunoglobulin replacement therapy for frequent or prolonged respiratory tract infections, which have not occurred in our patient up to the present.

Lymphocyte subset analysis in NBS has shown decreased T cells, with CD4+ T cells affected most severely [29]. This is consistent with our patient’s findings (Table 1). The elevated proportion of NK cells seen in our patient was also noted previously in 25 out of 40 patients [30].

The treatment of choice for SCID is most often HSCT. To date, however, there is limited experience with HSCT in patients with NBS [31]. Indications for attempting transplantation have included severe immunodeficiency and hematologic malignancy; pre-emptive HSCT has not been reported. Of particular concern has been the use of DNA-damaging chemotherapeutic conditioning regimens and the potential to induce *de novo* malignancies in patients with a pre-existing DNA repair deficit. There has been some success with reduced intensity pre-HSCT conditioning regimens, but studies to date are limited by their retrospective nature, small sample size and relatively short term follow-up.

## Conclusions

With widespread adoption of TREC screening for SCID, NBS may be identified prospectively, enabling the population incidence and natural history to be better understood. NBS has been reported primarily in Eastern Europeans, where founder mutations occur [28]. Our case expands our appreciation of the association of NBS with hydrocephalus and hydronephrosis and illustrates the utility of WES as applied to diagnose the underlying cause of idiopathic T lymphopenia found by TREC screening. There are more than 200 known genetic defects for primary immunodeficiency [19, 32], and testing of individual genes can be costly and time intensive. WES resulted in prompt detection of two nonsense mutations in *NBN* and definitive diagnosis in our patient, who presented with atypical features of NBS. Establishing the diagnosis allowed avoidance of live virus vaccines and exposure to ionizing radiation, as well as anticipatory monitoring for serious infections and malignancy.

Trimethoprim/sulfamethoxazole was started to prevent *Pneumocystis jiroveci* pneumonia; however he has not received immunoglobulin infusions or HSCT. Exome sequencing of the parents also established the *NBN* mutations as inherited versus de novo and allowed for accurate and informative counseling regarding the risk of future offspring inheriting both affected genes and having NBS. The parents were informed that other family members could be carriers of these same mutations.
